# The Relationship Between the Rationing of Nursing Care, Job Satisfaction, and Burnout Among Nurses in Northwestern Poland

**DOI:** 10.1155/jonm/7726865

**Published:** 2026-02-13

**Authors:** Małgorzata Szkup, Daria Schneider-Matyka, Kamila Rachubińska, Marzanna Stanisławska, Ewa Kupcewicz, Elżbieta Grochans, Anna Maria Cybulska

**Affiliations:** ^1^ Department of Social Nursing, Faculty of Health Sciences, Pomeranian Medical University in Szczecin, 48 Żołnierska St 71-210, Szczecin, Poland, pum.edu.pl; ^2^ Department of Nursing, Faculty of Health Sciences, Pomeranian Medical University in Szczecin, 48 Żołnierska St, Szczecin, 71-210, Poland, pum.edu.pl; ^3^ Department of Nursing, Collegium Medicum, University of Warmia and Mazury in Olsztyn, Olsztyn, Poland, uwm.edu.pl

**Keywords:** BERNCA-R, job burnout, job satisfaction, nursing care, rationing of nursing care

## Abstract

**Background:**

Rationing of nursing care, understood as the omission or inadequate performance of professional activities, is a phenomenon observed in medical care facilities around the world. It results not only in the risk of reducing patient trust and satisfaction with the care provided but also in adverse events that can significantly reduce patient safety.

**Aim:**

The aim of this study was to search for factors contributing to the rationing of care by Polish nurses.

**Material and Methods:**

This survey‐based study, which involved 528 nurses from northwestern Poland, was performed in 2023. It was performed using a tool of our own design and three standardized questionnaires: the Basel Extent of Rationing of Nursing Care–Revised (BERNCA‐R), the Satisfaction with Job Scale (SWJS), and the Maslach Burnout Inventory (MBI).

**Results:**

The average BERNCA‐R score was 1.52 points (SD = 0.95), which indicates a low level of rationing care by the surveyed nurses. Analysis of the SWJS results revealed that the respondents were rather dissatisfied with their work. The MBI confirmed high levels of burnout in all three subscales: emotional exhaustion (58.33%), depersonalization (55.3%), and personal accomplishment (83.5%). Linear regression analysis showed that in both univariate and multivariate models, the MBI emotional exhaustion subscale score, working 12 or 24 h shifts, and caring for a group of 11–20 patients were direct, independent predictors of rationing of nursing care as measured by the BERNCA‐R. Job satisfaction, due to its statistical significance verified by multivariate analysis, was an independent predictor (*p* = 0.004).

**Conclusions:**

The phenomenon of nursing care rationing is associated with increased workload, low job satisfaction, and emotional exhaustion.

## 1. Introduction

Rationing of nursing care (RONC) is a relatively new concept, first introduced by Schubert in 2007. It refers to necessary nursing tasks that nurses refuse or fail to do because of limited time, staffing level, or skill mix [1]. The idea of RONC has not yet been defined in detail. We still lack reliable research in this field, which results in differing opinions on this subject. Some authors, including Schubert, equate RONC with negligence, which takes the form of delay, careless performance, or omission of activities, thus emphasizing the negative nature of the phenomenon [2]. However, this concept can also be understood as the process of making decisions by nurses in the context of nursing care priorities [3]. Insufficient manpower, lack of skills, and time do not seem to be the only factors that can affect nursing care. RONC is a common problem in medical facilities in many countries around the world. Therefore, it is important to deepen knowledge in this area, especially in the face of evidence indicating the relationship between RONC and the quality of healthcare provided [4–7]. RONC may lead to adverse events, ultimately lowering the standard of care provided [8].

The findings of the present study can be interpreted within established frameworks of healthcare quality and nursing work organization. In particular, they are consistent with Donabedian’s structure–process–outcome model, in which structural conditions of nursing work, such as staffing and skill mix, shift patterns, and nurse‐to‐patient workload, shape care processes, including the extent to which necessary nursing activities are delivered or left undone (rationed). These care processes are, in turn, linked to outcomes such as patient safety, adverse events, and patient experience [9, 10]. From this perspective, RONC may be conceptualized primarily as a process‐level indicator of care quality, reflecting constraints in the organizational environment and potentially translating into downstream outcomes. In parallel, the Maslach conceptualization of burnout offers a complementary lens for understanding individual‐level mechanisms underlying RONC: emotional exhaustion, depersonalization, and reduced personal accomplishment may erode the cognitive and emotional resources required to sustain comprehensive nursing care, thereby increasing the likelihood of care rationing under conditions of high workload and time pressure [11, 12].

In the late 1990s, the International Hospital Outcomes Study (IHOS), the first international study to assess the organization of nursing care in hospitals and its impact on patient outcomes, was conducted. Subsequent studies such as the RONC in Switzerland (RICH), the Nurse Forecasting: Human Resources Planning in Nursing (RN4CAST), and the Rationing–Missed Nursing Care: An International and Multidimensional Problem (RANCARE) became the starting point for an international discussion on RONC, with the goal of planning strategies to reduce and prevent it at local, national, and international levels. Recent literature shows that RONC is a significant ethical issue in the healthcare system worldwide. Many researchers are trying to find out what contributes to this phenomenon in various clinical specialties and geographic areas. While Schubert et al. focused on the role of organizational factors, such as the type of department and hospital [1], others concluded that the greatest impact is exerted by the type of employment and teamwork. Staff shortages force nurses to prioritize interventions based on clinical judgment, which is inconsistent with the idea of holistic nursing care. This may adversely affect the quality of care and job satisfaction and increase the likelihood that patients’ needs will not be met, putting their health or lives at risk [13, 14]. A review of the literature indicates that RONC is determined by a number of factors, such as psychological issues, low job satisfaction [15–19], elevated stress levels [16], a higher risk of burnout [16, 19, 20], higher absenteeism from work [15], and increased staff turnover [21].

Job satisfaction is a multidimensional concept, referring to the realm of emotions and reflecting the interaction between expectations, work environment, as well as personal values and characteristics [22]. In light of research proving the unquestionable influence of job satisfaction on the quality of nursing care [23, 24], it seems reasonable to analyze the relationship between job satisfaction and RONC.

Occupational burnout, defined as a psychological state involving emotional exhaustion, depersonalization, and reduced personal accomplishment in people collaborating with others [25], has so far been poorly understood in terms of its impact (along with job satisfaction) on the extent of RONC in hospital settings [26]. Burnout has gained prominence as a social challenge that negatively affects the standard of nursing care [27]. Nevertheless, empirical evidence suggests that a supportive work environment and cohesive teamwork result in greater nurse engagement, which consequently improves the quality and safety of patient care [28]. Nurses showing high levels of RONC were significantly more emotionally exhausted and less effective at work [29, 30].

A review of the literature indicates that RONC is an international problem that adversely affects the quality of care provided and threatens patient safety. Despite many studies, there is still a significant information gap regarding the factors affecting RONC in different medical centers. Therefore, the purpose of this study was to identify possible contributors to RONC by analyzing its relationship with job burnout, job satisfaction, and characteristics of nurses and their workplace. The hypothesis was that increased burnout, lower job satisfaction, and work overload among nurses would likely exacerbate the incidence of RONC, consequently reducing the quality and safety of the nursing care provided.

## 2. Materials and Methods

### 2.1. Study Design and Settings

This survey‐based study, which involved nurses from northwestern Poland, was conducted from February to November 2023 using standardized research tools and a cross‐sectional design. Data were collected through paper questionnaires distributed among nurses in selected healthcare facilities, following prior approval from facility management and ward supervisors.

The questionnaires were distributed directly at the participants’ workplaces (hospital wards and other healthcare facilities) during nonclinical time and were completed anonymously and on a voluntary basis. Completed questionnaires were returned in sealed envelopes to ensure confidentiality. No questionnaires were sent to home addresses. This approach was intended to facilitate access to the target population while minimizing external pressure and promoting honest reporting on sensitive issues such as RONC and professional burnout.

The size of the study sample was determined on the basis of statistical data regarding professionally active nurses registered in the Szczecin Chamber of Nurses and Midwives and in the District Chamber of Nurses and Midwives in Koszalin in 2023. The confidence level was set at 95%, the maximum error was set at 5%, and the estimated fraction size was set at 0.5. According to the calculations, the required number of people who should participate in the study was 369.

**FIGURE 1:**
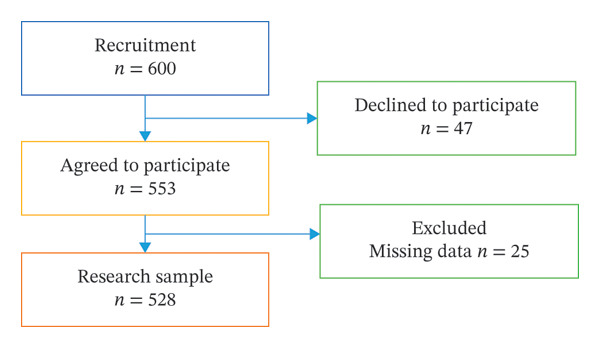
The study flowchart.

Medical facilities were selected using a purposive sampling approach, based on random invitation and the possibility of cooperation with senior management, which allowed access to nursing staff while maintaining diversity of clinical settings. A total of 600 nurses were invited to participate in the study. Ultimately, 528 correctly completed questionnaires were included in further analysis (response rate: 88%) (Figure 1).

### 2.2. Study Participants

Sets of questionnaires completed by 528 nurses were qualified for statistical analysis. The inclusion criteria for the study were as follows: informed and voluntary consent to participate in the study, the right to practice the profession, employment in a healthcare facility as a nurse for at least one year, and completing at least 95% of each questionnaire included in the survey. Questionnaires with more than 5% missing responses or completed in a way that prevented proper interpretation (e.g., marking several answers in single‐choice questions) were excluded from the study. The acceptance of questionnaires with minimal missing data was intended to maintain an adequate sample size while preserving data quality, which is a commonly applied approach in survey‐based studies using standardized instruments. Given the small proportion of missing responses, this procedure was unlikely to affect the reliability or validity of the results. Nurses with less than one year of experience were excluded from the study to ensure a more homogeneous sample in terms of work experience.

### 2.3. Ethical Considerations

The study protocol was approved by the Bioethics Committee of the Pomeranian Medical University in Szczecin, Poland. The study design was prepared in accordance with the Declaration of Helsinki. Each participant was informed in writing about the purpose and course of the study and gave written consent to participate in it. The respondents were also informed about the possibility of resigning from participation in the study at any stage without giving a reason. The study was conducted using a printed questionnaire with a cover letter explaining its purpose, as well as how to complete it and how long it would take. Completing the questionnaire at the workplace but outside of direct patient care hours was intended to reduce respondent burden and to promote thoughtful and reliable responses.

### 2.4. Research Tools

The author’s questionnaire was used to collect the necessary sociodemographic and work environment data, such as age, education, additional professional qualifications, type of employment, working hours, and the average number of patients under the care of the surveyed nurse. The average number of patients was self‐reported by the respondents, who were asked to indicate the average number of patients they usually care for, based on their own assessment of routine working conditions, rather than on a single last shift or a formally calculated average over a specified time period. In addition, we used standardized tools: the Basel Extent of Rationing of Nursing Care–Revised (BERNCA‐R), the Satisfaction with Job Scale (SWJS), and the Maslach Burnout Inventory (MBI).

The BERNCA‐R questionnaire was developed by Schubert et al. [1] to assess RONC in hospital settings. In our study, the Polish version of the questionnaire was used. The BERNCA‐R was adapted to Polish conditions by Uchmanowicz et al., with Cronbach’s alpha of 0.96 for a unidimensional scale [5]. The BERNCA‐R includes 32 items concerning basic nursing tasks and duties that may potentially go unperformed due to staffing shortages. These are divided into five categories: daily activities, care and support, rehabilitation and education, monitoring and safety, and documentation. Respondents rate their answers on a five‐point Likert scale (0: nonrequired, 1: never, 2: rarely, 3: sometimes, and 4: often) referring to their experiences during the preceding seven working days. The final score of the questionnaire is the average of the answers to these 32 questions. It thus ranges from 0 to 4 and can be interpreted analogously to the interpretation of a single question. Cronbach’s alpha and interitem correlations were employed to assess the internal consistency of the Polish BERNCA‐R questionnaire. The average total BERNCA‐R score was 1.9 points (SD = 0.74) on a scale ranging from 0 to 4. Cronbach’s alpha for the unidimensional scale was found to be 0.96. The mean interitem correlation was 0.4 (range: 0.1–0.84), indicating high internal consistency. A single‐factor solution showed stable loadings above 0.5 for nearly all items of the Polish BERNCA‐R questionnaire. The study utilizing the Polish BERNCA‐R questionnaire confirmed that the instrument is valid and reliable for investigating care rationing among Polish nurses [5].

The SWJS refers to the conscious assessment of one’s attitude toward work as a holistic, complex phenomenon, based on personal criteria. It consists of five items rated on a 7‐point Likert scale (1: I strongly disagree, i.e., I am definitely dissatisfied with a given aspect of my work; 2: I disagree; 3: I rather disagree; 4: It is difficult to say whether I agree or disagree; 5: I rather agree; 6: I agree; and 7: I strongly agree, i.e., I am definitely satisfied with a given aspect of my work). The SWJS score is the total number of points from the five questions (range: 5–35 points), and the higher the score, the greater the job satisfaction. For the SWJS, there are no standards that allow us to say what scores indicate high or low job satisfaction. However, it is possible to calculate the average number of points per question and interpret it according to the key for a single question. According to available data, the SWJS meets the criteria for reliability and relevance of a tool for measuring overall job satisfaction, showing high internal consistency (the Cronbach’s alpha coefficient is 0.80) [31].

The MBI is a 22‐item questionnaire, developed by Maslach et al. [32]. In our study, its Polish adaptation created by Pasikowski was used [33]. The tool encompasses three subscales: emotional exhaustion (9 items), depersonalization (5 items), and personal accomplishment (8 items; this subscale is interpreted reversely, the higher the score, the lower the perceived personal accomplishment). Each statement is rated on a 7‐point Likert scale (from 0 to 6). Results are computed separately for each subscale. Elevated scores on emotional exhaustion and depersonalization, combined with low scores on personal accomplishment, indicate the presence of burnout. The overall burnout index is derived from the average of the three subscales. High scores on emotional exhaustion and depersonalization, together with low scores on personal accomplishment, indicate occupational burnout in an individual [26]. There are norms for each of the subscales to determine whether a score indicates high, medium, or low levels of burnout (Cronbach’s alpha values for all three subscales were above 0.70).

### 2.5. Statistical Analysis

Analysis of quantitative variables was performed using descriptive statistics, including means, standard deviations, medians, quartiles, and minimum and maximum values. Categorical variables describing professional characteristics were analyzed using absolute frequencies and percentages. Univariate and multivariate linear regression analyses were conducted to assess the effects of potential predictors on quantitative outcome variables.

Prior to regression analyses, standard assumptions were evaluated. The normality of residuals was assessed by visual inspection of histograms and Q–Q plots, while homoscedasticity was examined using plots of residuals versus fitted values. No major violations of linear regression assumptions were observed. The results are presented as regression coefficients with 95% confidence intervals.

Multicollinearity among explanatory variables included in the multivariate models was assessed using variance inflation factors (VIFs), with values greater than five considered indicative of problematic multicollinearity. All VIF values were below the commonly accepted threshold of 5.0, indicating no evidence of multicollinearity. As the subjects per variable (SPV) index exceeded 10, no additional variable selection procedures were applied in the multivariate analyses.

A significance level of 0.05 was adopted; *p* values below this threshold were considered statistically significant. Cases with missing data were excluded from multivariable analyses using listwise deletion. All analyses were performed using *R* software (Version 4.3.2) [34].

## 3. Results

### 3.1. Characteristics of the Study Sample

A total of 528 nurses were qualified to take part in the study, with a mean age of 40.5 years (SD = 11.15) and work experience of 17.4 years (SD = 12.31). Most of them (55.49%) had a bachelor’s degree and additional professional qualifications (54.36%). The vast majority were employed under an employment contract (88.45%), worked a 12 or 24 h shift schedule (55.87%), and worked as a ward nurse (77.27%). The respondents provided care to patient groups varying in size from 1–10 (38.83%) to over 30 patients (17.99%) (Table 1).

**TABLE 1:** Characteristics of the study sample and working environment.

Parameter		*n*
Age (years)	Mean (SD)	40.5 (11.15)
	Median (quartiles)	44 (29–50)
	Range	23–66
	N	528

Education	Secondary	65 (12.31%)
	The first‐cycle higher education	293 (55.49%)
	The second‐ or third‐cycle higher education	170 (32.20%)

Additional professional qualifications	No	241 (45.64%)
	Yes	287 (54.36%)

Type of employment	Employment contract	467 (88.45%)
	Contractual or fee‐for‐task agreement	61 (11.55%)

Working hours	7 h 35 min	115 (21.78%)
	12 or 24 h	295 (55.87%)
	Mixed	118 (22.35%)

Years worked (years)	Mean (SD)	17.4 (12.31)
	Median (quartiles)	20 (4–28)
	Range	1–55

Position	Ward‐charge nurse or coordinating nurse	54 (10.23%)
	Ward nurse	408 (77.27%)
	Surgical nurse	66 (12.50%)

Average number of patients	1–10 patients	205 (38.83%)
	11–20 patients	144 (27.27%)
	21–30 patients	84 (15.91%)
	Over 30 patients	95 (17.99%)

#### 3.1.1. RONC, Job Satisfaction, and the Level of Burnout Among Polish Nurses

Analysis showed that the average total score on the BERNCA‐R questionnaire was 1.52 points (SD = 0.95). Thus, it can be concluded that the average frequency of RONC by the respondents was between the complete absence of such situations and their sporadic occurrence (Table 2).

**TABLE 2:** Characteristics of the BERNCA‐R score for the whole study sample.

BERNCA‐R (score)									
Score range	*N*	No data	Mean	SD	Median	Min	Max	Q1	Q3
0–4	528	0	1.52	0.95	1.47	0	3.97	0.75	2.19

*Note: N*, the total number of respondents; Min, minimum value; Max, maximum value; Q1, first quartile; Q3, third quartile.

Abbreviation: SD, standard deviation.

We analyzed the distribution of responses to individual BERNCA‐R questions in order to identify those aspects of nursing care that were most often neglected (rationed). These were as follows: talking to the patient or family (mean: 1.78), providing the patient with emotional or psychosocial support (1.73), getting to know the situation of individual patients and care plans at the start of the shift (1.72), monitoring the patient’s condition as closely as needed (1.71), and providing patients with information about upcoming tests or treatment (1.7) (Table 3).

**TABLE 3:** Analysis of the most frequently rationed activities according to the BERNCA‐R.

Question	Not required (0) (%)	Never (1) (%)	Rarely (2) (%)	Sometimes (3) (%)	Often (4) (%)	Mean
1. Could you not carry out necessary sponge baths for patients?	40.72	22.16	18.75	12.31	6.06	1.21
2. Could you not carry out the necessary partial sponge baths for patients?	35.23	29.17	19.51	12.69	3.41	1.2
3. Could you not carry out the necessary skin care for patients?	30.68	26.89	22.73	14.96	4.73	1.36
4. Could you not carry out the necessary oral hygiene for patients?	34.85	24.24	20.64	11.36	8.90	1.35
5. Could you not carry out the necessary dental hygiene for patients?	35.42	22.54	20.08	11.74	10.23	1.39
6. Could you not appropriately assist patients unable to eat independently?	32.95	28.03	21.40	12.69	4.92	1.29
7. Were you unable to mobilize patients as necessary, who had restricted mobility/motility or who were immobile?	27.27	24.43	27.08	14.77	6.44	1.49
8. Were you unable to change the position of patients who had restricted mobility/motility or who were immobile?	27.46	23.86	25.19	17.23	6.25	1.51
9. Could you not change, in an adequate time period, patients’ bed linen strongly soiled with urine, stool, or vomit?	28.79	26.89	23.11	14.58	6.63	1.43
10. Could you not offer emotional or psychosocial support to a patient, even though you felt it was necessary, e.g., dealing with insecurities and fear of his/her illness and feelings of dependency?	20.27	24.43	27.08	17.99	10.23	1.73
11. Could you not have a necessary conversation with a patient or his/her family?	18.75	25.19	25.00	21.40	9.66	1.78
12. Could you not inform patients sufficiently about imminent tests or planned therapies?	19.89	25.00	27.46	20.64	7.01	1.7
13. Could you not carry out toilet or continence training of patients and therefore had to put them in diapers?	33.52	20.08	22.54	16.67	7.20	1.44
14. Could you not carry out toilet or continence training of patients and therefore had to insert a permanent catheter?	34.28	20.83	22.16	15.91	6.82	1.4
15. Could you not carry out activating or rehabilitating care for patients?	3.95	18.94	23.30	13.45	11.36	1.51
16. Were you unable to train and/or educate patients and/or their families, e.g., insulin injection, behavior, or coping with illness‐specific symptoms (hypoglycemia and dyspnea)?	31.63	20.83	22.92	14.02	10.61	1.51
17. Were you unable to fully prepare patients or their families for hospital discharge?	30.68	22.35	21.59	16.48	8.90	1.51
18. Were you unable to monitor patients as closely as had been prescribed by their physicians?	21.40	27.08	25.19	17.05	9.28	1.66
19. Were you unable to monitor patients as closely as you felt was necessary?	18.94	28.60	25.19	16.86	10.42	1.71
20. Were you unable to watch confused patients carefully enough and therefore had to restrain them?	29.36	21.21	23.48	17.42	8.52	1.55
21. Were you unable to watch confused patients carefully enough and therefore had to sedate them?	28.79	24.05	20.45	16.48	10.23	1.55
22. Were you forced to delay necessary measures to assist patients with unforeseen, sudden or acute changes in status, because the physicians called arrived very late?	27.84	28.60	21.21	13.83	8.52	1.47
23. Were you unable to administer a prescribed medication and/or infusion at the recommended time?	23.30	27.08	24.62	17.99	7.01	1.58
24. Could you not change/apply the necessary wound dressings for patients?	20.83	32.58	23.48	17.23	5.87	1.55
25. Could you not prepare patients for tests or therapies?	23.67	29.36	27.84	14.58	4.55	1.47
26. Did you have to keep patients who had rung for a nurse waiting longer than 5 min?	29.17	28.03	19.89	16.10	6.82	1.43
27. Could you not carry out adequate hand hygiene?	19.13	38.07	23.11	12.69	7.01	1.5
28. Could you not comply with the necessary disinfection measures?	17.99	36.36	25.00	13.83	6.82	1.55
29. Did you not have enough time to study the care plans to inform yourself about the patient situation at the beginning of your shift?	18.94	26.52	27.84	17.42	9.28	1.72
30. Were you unable to ascertain needs assessments for newly admitted patients?	21.02	25.19	28.03	17.42	8.33	1.67
31. Were you unable to set up patients’ care plans?	23.48	25.19	25.00	18.56	7.77	1.62
32. Could you not sufficiently document and evaluate the care carried out for patients?	18.75	29.73	23.67	19.89	7.95	1.69

The average score for job satisfaction as measured by the SWJS was 16.63 points, with an average of 3.33 points per question. The rounded result was 3, meaning that the respondents were rather dissatisfied with their work (Table 4).

**TABLE 4:** The respondents’ job satisfaction according to the SWJS.

SWJS (score)										
Score range	*N*	No data	Mean	SD	Average score per question	Median	Min	Max	Q1	Q3
5–35	528	0	16.63	5.95	3.33	16	5	35	13	20

*Note: N*, the total number of respondents; Min, minimum value; Max, maximum value; Q1, first quartile; Q3, third quartile.

Abbreviation: SD, standard deviation.

For the MBI subscales, the largest proportion of respondents (58.33%) indicated a high level, and only 10.8% reported a low level of emotional exhaustion. The results for the subscale of depersonalization were similar: 55.3% had a high level, and 11.93% had a low level of depersonalization. The biggest differences were noted for the personal accomplishment subscale: 83.5% of the respondents had a high feeling, and 3.6% had a low feeling of a lack of personal achievement (Table 5).

**TABLE 5:** The MBI subscales.

MBI	Score	Interpretation	*n*	%
Emotional exhaustion	0–16	Low	57	10.80
	17–26	Medium	163	30.87
	Over 26	High	308	58.33

Depersonalization	0–6	Low	63	11.93
	7–12	Medium	173	32.77
	Over 12	High	292	55.30

Personal accomplishment	0–31	High	441	83.52
	32–38	Medium	68	12.88
	Over 38	Low	19	3.60

*Note: n*, number of individuals; %, percent of individuals.

### 3.2. The Influence of Job Satisfaction According to the SWJS and Job Burnout According to the MBI on the Extent of RONC According to the BERNCA‐R

Univariate (or single‐variable) linear regression analysis showed that each of the three aspects of job burnout included in the MBI had an impact on the extent of RONC as measured by the BERNCA‐R. Each point on the MBI emotional exhaustion subscale raised the BERNCA‐R score by an average of 0.024 points, and each point on the MBI depersonalization subscale raised the BERNCA‐R score by an average of 0.035 points, while each point on the MBI personal accomplishment subscale reduced the BERNCA‐R score by an average of 0.028 points. Other statistically significant determinants (< 0.001) were working 12 or 24 h shifts (which raised the BERNCA‐R score by an average of 0.477 points compared to working 7 h and 35 min shifts) and caring for 11–20 patients (which raised the BERNCA‐R score by an average of 0.349 points compared to caring for 1–10 patients). A similar relationship was also observed in relation to the position held: working as a ward nurse raised the BERNCA‐R score by an average of 0.302 points compared to working as a ward‐charge or a coordinating nurse (*p* = 0.028).

Multivariate linear regression analysis revealed the following relationships: each point on the SWJS raised the BERNCA score by an average of 0.021 points, each point on the MBI emotional exhaustion subscale raised the BERNCA score by an average of 0.019 points, working 12 or 24 h shifts raised the BERNCA‐R score by an average of 0.384 points (compared to working 7 h 35 min), and caring for 11–20 patients raised the BERNCA score by an average of 0.338 points (compared to caring for 1–10 patients).

In both univariate and multivariate models, the MBI emotional exhaustion subscale score, working 12 or 24 h shifts, and caring for a group of 11–20 patients were direct, independent predictors of RONC as measured by the BERNCA‐R. Job satisfaction, due to its statistical significance verified by multivariate analysis, was an independent predictor (*p* = 0.004). This result, however, was biased by confounding factors, which made it insignificant in univariate analysis. Other factors, such as depersonalization, reduced personal accomplishment, and working as a ward nurse, were indirect predictors, significant only in univariate analyses and, therefore, probably having no direct but only an apparent effect on RONC (Table 6).

**TABLE 6:** The effect of job satisfaction, burnout, and professional characteristics on the extent of RONC.

Variable		Univariate model				Multivariate model^#^			
		Parameter	95% CI		*p*	Parameter	95% CI		*p*
SWJS (score)		0.001	−0.013	0.014	0.929	0.021	0.007	0.036	0.004^∗^
MBI: emotional exhaustion (score)		0.024	0.015	0.033	< 0.001^∗^	0.019	0.003	0.034	0.017^∗^
MBI: depersonalization (score)		0.035	0.019	0.051	< 0.001^∗^	0.006	−0.019	0.03	0.648
MBI: personal accomplishment (score)		−0.028	−0.039	−0.017	< 0.001^∗^	−0.011	−0.028	0.005	0.176

Age (years)		−0.002	−0.009	0.005	0.602				

Education	Secondary	ref.				ref.			
	The first‐cycle higher education	0.136	−0.12	0.392	0.298	0.094	−0.177	0.365	0.498
	The second‐ or third‐cycle higher education	0.035	−0.237	0.307	0.801	0.102	−0.182	0.385	0.482

Additional professional qualifications	No	ref.				ref.			
	Yes	−0.046	−0.209	0.117	0.58	−0.068	−0.266	0.131	0.505

Type of employment	Employment contract	ref.				ref.			
	Contractual or fee‐for‐task agreement	−0.234	−0.488	0.02	0.071	−0.132	−0.396	0.132	0.328

Working hours	7 h 35 min	ref.				ref.			
	12 or 24 h	0.477	0.276	0.678	< 0.001^∗^	0.384	0.156	0.612	0.001^∗^
	Mixed	0.165	−0.075	0.404	0.178	0.182	−0.081	0.446	0.176

Years worked (years)		−0.001	−0.007	0.006	0.819	0.003	−0.006	0.011	0.562

Position	Ward‐charge or coordinating nurse	ref.				ref.			
	Ward nurse	0.302	0.033	0.571	0.028^∗^	0.048	−0.24	0.336	0.743
	Surgical nurse	0.04	−0.301	0.38	0.819	−0.12	−0.46	0.22	0.49

Average number of patients	1–10 patients	ref.				ref.			
	11–20 patients	0.349	0.148	0.551	0.001^∗^	0.338	0.143	0.532	0.001^∗^
	21–30 patients	0.209	−0.031	0.449	0.088	0.192	−0.043	0.428	0.11
	More than 30 patients	0.101	−0.129	0.331	0.39	0.19	−0.039	0.418	0.105

^∗^Statistical significance (*p* < 0.05).

^#^Model: SWJS, MBI, education, additional professional qualifications, type of employment, working hours, years worked, position, and average number of patients.

## 4. Discussion

There are numerous studies worldwide providing scientific evidence for the relationship between inadequate nursing care and its negative consequences for patients, including higher morbidity and mortality, lower satisfaction with care, as well as increased costs for healthcare facilities [2, 35–38]. One of the mechanisms underlying this relationship may be RONC, understood as the failure to perform or the inadequate performance of nursing tasks due to limited resources. Accordingly, the present study focused on identifying factors that may contribute to RONC among Polish nurses.

Professional burnout, which is a relatively common phenomenon among nurses in Poland, may have multiple negative consequences for patients, healthcare organizations, and the nurses themselves. It is typically manifested by reduced engagement and conscientiousness, lower work efficiency, decreased quality of care, diminished effort, and a higher frequency of errors. Previous studies indicate that professional burnout can be considered an independent predictor of medical errors [38], higher rates of nosocomial infections [39], adverse events [40], as well as negligence and unethical behavior among healthcare staff [41]. In the present study, a high level of emotional exhaustion, a core component of professional burnout according to Maslach et al. [28], was identified as a direct and independent predictor of RONC among Polish nurses.

These findings are consistent with the results of a study conducted by Uchmanowicz et al. in public hospitals in Poland, which confirmed a relationship between RONC and all three dimensions of burnout described by Maslach et al., namely, emotional exhaustion, depersonalization, and a reduced sense of personal accomplishment [32]. Among these dimensions, emotional exhaustion has been shown to exert the strongest influence on the overall level of burnout [26]. Our results further align with evidence reported by Uchmanowicz et al., demonstrating that higher levels of burnout and lower job satisfaction were associated with increased RONC while highlighting the managerial relevance of addressing burnout as a potential target for improving nurse outcomes and care delivery [26].

Kalisch et al. emphasized the moral burden experienced by nurses as a result of RONC. This burden carries significant emotional implications, as the omission of care often conflicts with nurses’ professional values and beliefs, potentially distorting their self‐perception and sense of professional role within the healthcare system [42].

In the present study, the nursing activities most frequently reported as rationed were those related to communication with patients, including conversations with patients or their families, the provision of emotional or psychosocial support, and informing patients about upcoming diagnostic tests or treatment. According to Jones et al., burnout has a particularly strong impact on communication between healthcare personnel and patients, leading to weakened interpersonal contact and reduced patient access to essential health‐related information [2].

Increased levels of burnout are widely recognized as a factor contributing to reduced job satisfaction [26, 43, 44]. Our findings confirm that low job satisfaction can be regarded as an independent predictor of RONC among nurses. Similar negative associations between job satisfaction and RONC have been reported by other authors [2, 13, 15]. At the same time, RONC may not only result from low job satisfaction but may also contribute to its further decline [45]. Mandal et al. [4] demonstrated that nurses who ration care report lower job satisfaction. Other studies have pointed to additional consequences of RONC, including moral distress, feelings of guilt and worry [46, 47], as well as an increased intention to leave the profession and higher staff turnover among nurses experiencing RONC [26, 48, 49].

Furthermore, a systematic review by Stemmer et al. concluded that unfinished or rationed nursing care is frequently associated with reduced job satisfaction and, less consistently, with burnout, while also being linked to intention to leave in the available studies [50]. This broader international evidence supports our findings that job satisfaction and emotional exhaustion are relevant correlates of RONC while simultaneously highlighting persistent limitations in the literature, such as reliance on self‐reported measures and predominantly single‐country study designs.

In our study, RONC was additionally associated with high workload, reflected by caring for larger patient groups (11–20 patients compared to 1–10 patients) and working extended shifts of 12 or 24 h rather than standard‐length shifts. Increased workload may place nurses in situations where, despite sustained effort, they are unable to meet all patient needs. This, in turn, may contribute to negative self‐evaluation of job performance and further reductions in job satisfaction [15, 51].

The extent of the RONC problem varies across countries and has not yet been fully elucidated. The IHOS, which included more than 43,000 nurses from over 700 hospitals in the United States, Canada, England, Scotland, and Germany, demonstrated that only 30%–40% of nurses perceived staffing levels in their workplace as sufficient to ensure high‐quality nursing care. A substantial proportion of participants (10%–54%, depending on the country and nursing task) reported that activities considered indicators of appropriate nursing care, such as patient education, oral hygiene, or communication, were not performed during their most recent shift [52]. Similar findings were reported in Switzerland, where 30% of nurses experienced difficulties in maintaining appropriate communication and ensuring patient comfort [53], and in Great Britain, where 64% of nurses declared being unable to complete basic nursing tasks due to time constraints [54].

An interesting and noteworthy finding of the present study is the coexistence of low job satisfaction and a high rate of burnout with a relatively low level of self‐reported rationing of nursing care. This apparent discrepancy has also been noted in previous studies and may reflect strong professional values and ethical commitment among nurses, leading them to prioritize patient care despite significant personal strain. Qualitative and conceptual analyses suggest that nurses may engage in compensatory behaviors, such as working faster, skipping breaks, or extending unpaid effort, in order to minimize omissions in care, even when experiencing emotional exhaustion and reduced well‐being [42, 46, 55]. Moreover, cultural norms and expectations regarding professional responsibility may influence how nurses perceive and report care rationing. In some contexts, admitting to missed or unfinished care may be perceived as a professional failure, resulting in systematic underreporting of RONC, particularly in self‐administered questionnaires. Previous research has highlighted that self‐reported measures of rationed or missed care are susceptible to social desirability bias, response normalization, and a floor effect, especially in settings where nurses are accustomed to chronic understaffing and high workloads [45, 56]. Similar patterns have been observed in other studies conducted in Poland and across Europe, where high levels of burnout coexisted with modest average scores of rationed nursing care. For example, Szkup et al. and Uchmanowicz et al. reported that although emotional exhaustion was strongly associated with RONC, the overall frequency of reported care rationing remained relatively low, suggesting that burnout may initially manifest at the psychological level before translating into observable omissions of care. This interpretation is further supported by international evidence indicating that emotional exhaustion often precedes behavioral withdrawal and missed care [26, 52].

The use of retrospective, self‐reported data represents a potential limitation of the present study, as responses may be subjective and not fully reflect actual practice [56]. Although anonymity was ensured, which may reduce socially desirable responding, it is not possible to determine the individual strategies or evaluation criteria employed by nurses when completing the questionnaires [1]. This limitation may have contributed to an underestimation of the true scale of RONC.

### 4.1. Implications

RONC is an international problem that adversely affects the quality of care provided and threatens patient safety. It is a priority for those managing medical facilities to find effective forms of employment and minimize excessive workload of nursing staff. Nurses should be aware of the potential consequences of RONC and develop effective strategies to reduce it. It is crucial that adverse events are reported and that detailed analyses of errors and the consequences of not performing all nursing tasks are carried out. Effective communication between nursing staff and managers of medical facilities is also important.

It is worth conducting further research to identify and understand other determinants of RONC, such as those related to work environment, workload or organizational support. The knowledge thus gained will enable the development of targeted interventions and strategies for nursing care and, consequently, patient safety.

### 4.2. Limitations and Strengths of the Study

The main strength of the study is the large number of respondents. Another strength is the effort made to ensure random sampling and a cross‐sectional observational design. Overall, the findings support the need for a comprehensive approach to the RONC, occupational stress, and their contributing factors.

The study also has several limitations. First, it included only nurses working in hospitals in northwestern Poland, which may limit the generalizability of the findings. Moreover, not all factors related to nurses, patients, and the organization of nursing work that may contribute to RONC were considered. Future research should therefore include healthcare facilities providing specialized care across different disciplines and European countries, as well as additional organizational and individual‐level variables.

Another limitation is the use of a self‐reported questionnaire, which may have introduced reporting bias, including socially desirable responding, particularly with regard to sensitive issues such as missed or rationed care. Consequently, the prevalence of RONC may have been underestimated.

An additional limitation of the present study is its cross‐sectional design, which precludes causal inference regarding the observed relationships between burnout, job satisfaction, workload, and RONC. Although several variables emerged as independent predictors, the directionality of these associations cannot be determined, and reciprocal or bidirectional relationships cannot be excluded. Accordingly, future studies employing longitudinal, interventional, or mixed‐methods designs are warranted to better capture temporal relationships and to evaluate organizational and managerial strategies.

Finally, although the present analyses focused on identifying independent predictors of RONC, future research may benefit from examining more complex pathways. In particular, job satisfaction and burnout may act as potential mediators or moderators in the relationship between workload and RONC. Testing such mechanisms would require longitudinal or interventional designs and was beyond the scope of the present cross‐sectional study.

## 5. Conclusions

1. RONC does not seem to be a common phenomenon among Polish nurses.
2. Polish nurses are rather dissatisfied with their work. Their level of burnout is high and is associated with a strong sense of reduced personal accomplishment, emotional exhaustion, and depersonalization.
3. It appears that the phenomenon of RONC is associated with increased workload. Caring for a group of 11–20 patients, working 12 or 24 h shifts, and a high level of emotional exhaustion are direct, independent predictors of RONC.
4. Low job satisfaction is an element that can be considered an independent predictor of increased RONC.

## Author Contributions

Conceptualization: Małgorzata Szkup and Anna Maria Cybulska. Methodology: Daria Schneider‐Matyka and Kamila Rachubińska. Formal analysis: Małgorzata Szkup and Anna Maria Cybulska. Investigation: Ewa Kupcewicz and Marzanna Stanisławska. Writing–original draft preparation: Małgorzata Szkup and Anna Maria Cybulska. Writing–review and editing: Małgorzata Szkup and Anna Maria Cybulska. Visualization: Daria Schneider‐Matyka and Kamila Rachubińska. Supervision: Ewa Kupcewicz and Elżbieta Grochans. Project administration: Elżbieta Grochans.

## Funding

No funding was received for this research.

## Disclosure

All authors have read and agreed to the published version of the manuscript.

## Ethics Statement

The study was conducted in accordance with the Declaration of Helsinki and was approved by the Bioethics Committee of the Pomeranian Medical University in Szczecin (KB.006.52.2024).

## Consent

All participants were fully informed that their anonymity is assured, why the research is being conducted, how their data will be used, and if there are any risks associated.

## Conflicts of Interest

The authors declare no conflicts of interest.

## Data Availability

The data that support the findings of this study are available from the corresponding author upon reasonable request.
